# Assessment of Native Myocardial T1 Mapping for Early Detection of Anthracycline-Induced Cardiotoxicity in Patients with Cancer: a Systematic Review and Meta-analysis

**DOI:** 10.1007/s12012-024-09866-1

**Published:** 2024-05-03

**Authors:** Amira A. Mohamed, Layla Y. Elmancy, Sara M. Abulola, Sara A. Al-Qattan, Mohamed Izham Mohamed Ibrahim, Zaid H. Maayah

**Affiliations:** 1 Department of Pharmaceutical Sciences, College of Pharmacy, QU Health, Qatar University, 2713 Doha, Qatar; 2 Clinical Pharmacy and Practice Department, College of Pharmacy, QU Health, Qatar University, P.O. Box 2713, Doha, Qatar

**Keywords:** Cancer, Anthracycline, T1 mapping, Inflammation, Fibrosis

## Abstract

Anthracycline antibiotic is one of the most effective anti-tumor drugs used to manage certain types of breast cancers, lymphomas, and leukemias. However, anthracyclines induce a dose-dependent cardiotoxicity that may progress to heart failure. Thus, using a sensitive predictor of early cardiac dysfunction in patients treated with anthracyclines can help detect subclinical cardiac dysfunction early and help initiate interventions to protect these patients. Among parameters of myocardial measure, cardiac magnetic resonance (CMR)-measured native myocardial T1 mapping is considered a sensitive and accurate quantitative measure of early subclinical cardiac changes, particularly cardiac inflammation and fibrosis. However, to understand the quality and the validity of the current evidence supporting the use of these measures in patients treated with anthracyclines, we aimed to conduct a systematic review of clinical studies of this measure to detect early myocardial changes in cancer patients treated with anthracyclines. The primary outcome was the level of native T1 mapping. We performed fixed-effects meta-analyses and assessed certainty in effect estimates. Of the 1780 publications reviewed (till 2022), 23 were retrieved, and 9 articles met the inclusion criteria. Our study showed that exposure to anthracycline was associated with a significant elevation of native myocardial T1 mapping from baseline (95% CI 0.1121 to 0.5802; *p* = 0.0037) as well as compared to healthy control patients (95% CI 0.2925 to 0.7448; *p* < 0.0001). No significant publication bias was noted on the assessment of the funnel plot and Egger’s test. According to the *Q *test, there was no significant heterogeneity in the included studies (*I*^2^ = 0.0000% versus healthy controls and *I*^2^ = 14.0666% versus baseline). Overall, our study suggests that native myocardial T1 mapping is useful for detecting anthracycline-induced cardiotoxicity in patients with cancer.

**Supplementary Information:**

The online version contains supplementary material available at 10.1007/s12012-024-09866-1.

## Introduction

Anthracycline antibiotic is one of the most effective anti-tumor drugs used to manage certain types of breast cancers, lymphomas, and leukemias [1]. However, anthracyclines induce a dose-dependent cardiotoxicity that may progress to heart failure [2]. In addition, in patients with depressed heart function at baseline, lower dosages of anthracyclines are used in full awareness that antineoplastic activity is dose dependent [3]. Furthermore, clinical monitoring of left ventricular (LV) function during chemotherapy has little proven value since many patients eventually progress to heart failure from global depression of cardiac ejection fraction after chemotherapy has been halted or completed [4]. Interestingly, while the incidence of cancer has increased significantly worldwide over the past decade, the rate of survival has increased due to improvements in diagnoses and therapies. However, the number of patients at risk for anthracycline cardiotoxicity is obviously increasing [5, 6].

Equally troubling is the fact that anthracycline-mediated decline in LV systolic function does not completely normalize when conventional heart failure therapy is initiated 4 months after an initial decline in left ventricular ejection fraction (LVEF) in patients with or without heart failure symptoms [7]. Additionally, LVEF does not completely recover in one-third of patients when heart failure therapy was initiated within 1–2 months after cardiac insult [7]. Thus, there is an obvious clinical need to detect anthracycline cardiotoxicity before the initial decline in LVEF [8]. Indeed, using a sensitive predictor of early cardiac dysfunction in patients treated with anthracyclines can help in the early detection of subclinical cardiac dysfunction and help in initiating interventions to protect these patients [8]. Given the cardiotoxicity in patients treated with anthracycline, protecting the heart from this side-effect would improve the risk-to-benefit ratio of anthracycline chemotherapy and lead to better quality and duration of post-chemotherapy life.

Among parameters of myocardial measure, cardiac magnetic resonance (CMR)-measured native myocardial T1 mapping is considered a sensitive and accurate quantitative measure of early subclinical cardiac changes [9, 10]. Unlike other myocardial measures, native myocardial T1 mapping can detect the changes at the myocardial tissue level likely to precede functional changes [10]. However, to understand the quality and the validity of the current evidence supporting the use of these measures in patients treated with anthracyclines, it is imperative to perform a systematic review and meta-analysis of studies investigating CMR-measured native myocardial T1 mapping as a predictor of anthracycline-related early subclinical cardiac dysfunction. Thus, we aim to synthesize results from clinical studies in an attempt to provide a conclusive answer on the validity of these measures to detect early myocardial changes in cancer patients treated with anthracyclines.

## Methods

### Search Strategy

This study followed the Cochrane Review Protocol (https://training.cochrane.org/handbook). In addition, we registered our study in PROSPERO—International Prospective Register of Systematic Reviews (CRD42022331267). Our study is based on a systematic review of the clinical trials on the role of native myocardial T1 mapping in the early detection of anthracycline-induced cardiotoxicity in patients with cancer. The systematic review of this project was performed based on the Preferred Reporting Items for Systematic Reviews and Meta-Analyses (PRISMA) protocol. The following databases were used to extract relevant data: PubMed, Embase, Web of Sciences, and Scopus. The search terms were created according to the population, intervention, comparison, and outcome (PICO) question: “*What is the value of native myocardial T1 mapping for the early detection of anthracycline-induced cardiotoxicity in patients with cancer?*”. The key search term for the population was according to the domain “cancer,” whereas for the intervention was “anthracyclines.” The comparator terms include “myocardial T1 mapping,” and finally, the outcomes were mainly based on the results from the clinical studies (Supplementary Table 1) [11–19].

### Selection Criteria

All studies were selected according to our inclusion criteria which include the articles in English language, peer-reviewed articles and reporting clinical studies on myocardial T1 mapping after exposure to any cumulative dose of anthracyclines used in adult cancer patients and compared the outcome with that of a healthy control group who were not treated with anthracyclines or baseline till November 2022. Our exclusion criteria include clinical studies where patients exposed to multiple cardiotoxic pharmacotherapies or less than 50% of patients’ cohort had anthracycline, if there is no baseline or healthy control, case reports, conference abstracts, duplicate studies, or reviews as well as non-clinical studies, such as animal studies and lab studies. The authors independently evaluated the studies to ensure that they matched the inclusion criteria. In cases of discrepancy, general agreement was attained by discussion between the authors.

### Data Collection

The authors uploaded search results from PubMed, Embase, Web of Sciences, and Scopus to the Rayyan software program (https://www.rayyan.ai/). The Rayyan software helped remove duplications and manually screened searched abstracts and articles. The data extracted from the selected clinical studies includes information about the number of patients, year and country, study population, sex, age, study design, intervention and outcome. The PRISMA protocol was used to analyze the data qualitatively and descriptively.

### Quality Assessment

The quality assessment of our collected reports was performed by all authors independently and any disagreement between them was resolved by consensus. The Joanna Briggs Institute (JBI) critical appraisal checklist was used to assess the quality of the studies.

### Statistical Analysis

Publication bias was assessed with visual inspection of funnel plot analysis, and Egger’s test was used to determine the funnel plot asymmetry. The meta-analysis was done using jamovi version 2.4.11 (Edelsbrunner, Peter (2017-03-23). The data were presented using the forest plot. Also, the data were pooled to calculate the standardized mean difference with a 95% confidence interval using a fixed-effects model. In our study, we utilized a standardized mean difference as our meta-analysis assessed native T1 mapping in various studies that have been measured differently. Standardized mean difference is used to measure the effect size of the study. Also, it is important to standardize the results of the studies to a uniform scale before they can be combined in this meta-analysis. A *p* value < 0.05 is considered significant. Heterogeneity was assessed using the *I*^2^ parameter by Chi-squared test (high heterogeneity if *I*^2^ is ≥ 50%). Leave-one-out sensitivity analysis was also performed to assess the level of heterogeneity.

## Results

### Study Selection

**Fig. 1:**
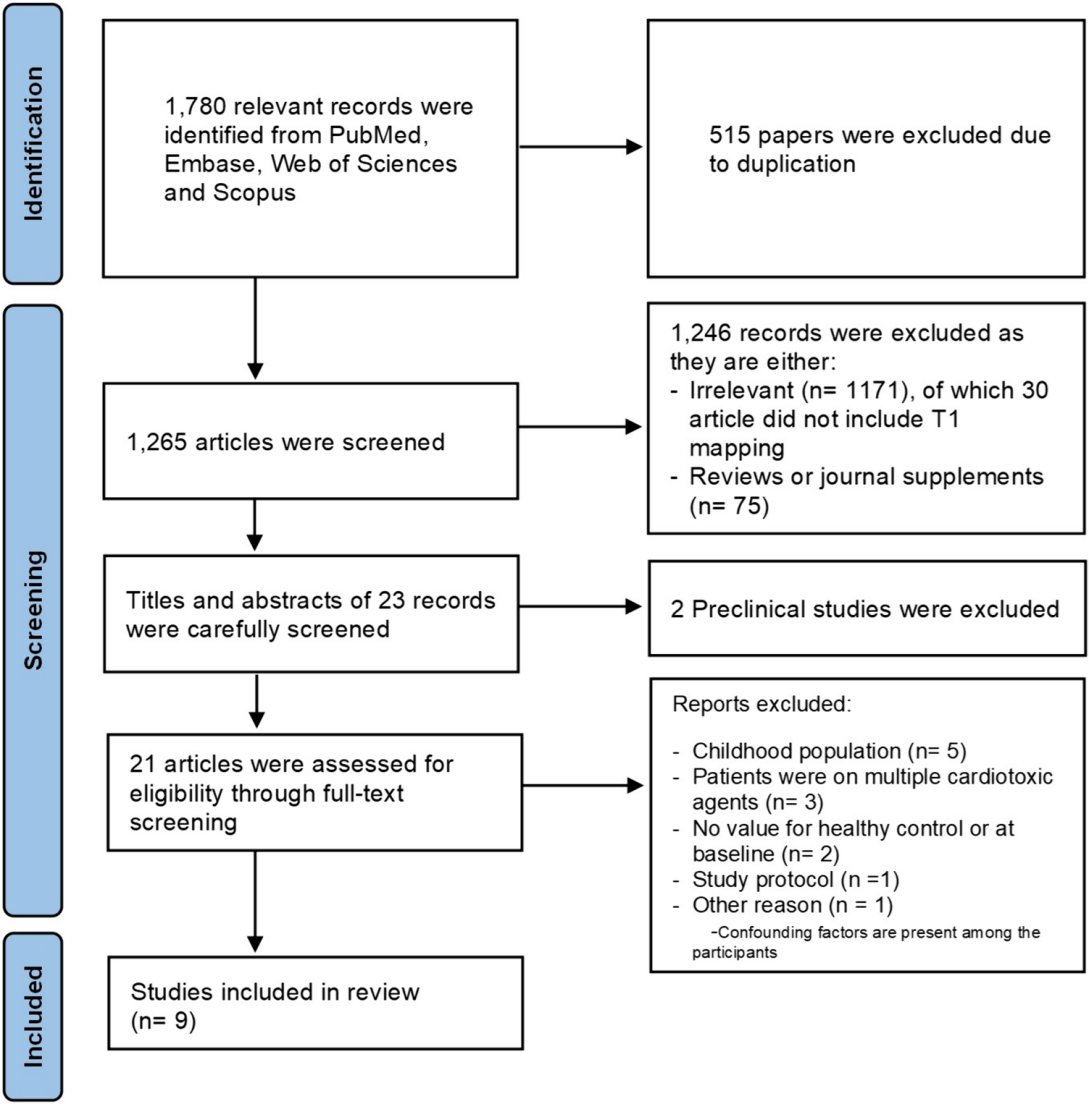
PRISMA flow diagram for studies evaluating T1 mapping in patients with anthracycline compared to healthy control or baseline

Our systematic review resulted in 1780 relevant records identified using PubMed, Embase, Web of Sciences, and Scopus. Of these, 515 papers were excluded due to duplications. After careful screening, 1246 articles were excluded as they were irrelevant, reviews, case reports, or conference abstracts or did not include myocardial T1 mapping. This resulted in 23 articles that were carefully screened. Of these, 13 studies were excluded for meeting the exclusion criteria. This includes two preclinical studies, five childhood population studies, three clinical studies where patients were exposed to multiple cardiotoxic pharmacotherapies, 2 studies with no baseline or healthy control group, and one study protocol. While ten studies met our initial selection criteria, Jordan et al. 2016 [20] was excluded as 31% of cancer patients already exhibited low glomerular filtration rate (GFR), two different contrast agents were used, and the presence of cardiovascular co-morbidities in some patients. Hence, nine articles were included in the meta-analysis with the data evaluating native myocardial T1 mapping for the detection of anthracycline-induced cardiotoxicity in cancer patients. The PRISMA flow diagram presents our systematic review results (Fig. 1).

### Study Characteristics

While the majority of patients were diagnosed with breast cancer, other types of cancer were present in a few studies, including soft tissue sarcoma and hematological malignancies, such as lymphomas (Table 1). In these studies, the patients' ages ranged between 47 and 59 years old (Table 1). Notably, the majority consisted of females, with three studies exclusively involving female patients and the remaining studies having over 45% female population (Table 1). In all studies, all patients received anthracycline-based chemotherapy regimens, with one study also including radiotherapy for all patients (Table 1). Lastly, while the LVEF in cancer patients treated with anthracyclines slightly decreased from baseline and compared to healthy controls (Supplementary Table 2 and 3), they were all within the normal range, suggesting that anthracyclines trigger subclinical cardiotoxicity in cancer patients among included studies.

**Table 1:** The effect of anthracycline on myocardial T1 mapping in clinical studies

Study	Country	Study population	Study design	Intervention	Sex	Age (years, mean ± SD)	Outcome
(Altaha et al. 2020)	Canada	Breast cancer women (*n* = 20) and their healthy control women (*n* = 30)	Single-center quasi-experimental study	Anthracycline-based therapy (303.85 ± 16.47 mg/m^2^). Only 1 patient had completed radiation therapy	100% female for cancer patients. 60% female for healthy controls	53.25 ± 7.6 for cancer women. 46.0 ± 13.7 for healthy controls	T1 mapping was higher in breast cancer patients compared to control
(Barbosa et al. 2021)	Brazil	Long-term adult survivors of non-Hodgkin lymphoma previously treated with anthracycline (*n* = 18) and their matched healthy control (*n* = 17)	Single-center quasi-experimental study	Anthracycline containing therapy (400 mg/m^2^ (IQR: 225–400 mg/m^2^). Two (11.1%) patients received mediastinal radiotherapy at the time of treatment	23% female for cancer patients. 53% female for healthy controls	57.6 (± 14.7) for cancer patients. 48.1 ± 12.2 for healthy controls	No significant change in myocardial T1 mapping
(Costello et al. 2019)	Australia	Newly diagnosed breast cancer patients (*n* = 27)	Single-center quasi-experimental study	Anthracycline-based therapy	–	–	A significant elevation of myocardial T1 mapping across all participants
(Kirkham et al. 2021)	Canada	Breast cancer women received anthracycline treatment ~ 1 yr earlier (*n* = 16), women with breast cancer who had not yet received treatment (*n* = 16), and age-matched and body mass index (BMI)-matched healthy control women (*n* = 16)	Single-center quasi-experimental study	Anthracycline-based therapy. Patients were also exposed to radiotherapy (11 (69%) left, 5 (31%) right)	100% female	56 ± 10 for cancer patients. 56 ± 10 for healthy controls	Myocardial T1 mapping was only elevated in women with breast cancer who had received anthracycline treatment
(Harries et al. 2021)	UK	Hematological malignancy (*n* = 33; 73%), breast cancer (*n* = 10; 22%), or others (*n* = 2; 4%) exposed to anthracycline (*n* = 45 in total) and compared to healthy control subjects of similar age and sex (*n* = 45)	Single-center quasi-experimental observational study	Anthracycline-based therapy. (237 ± 83 mg/m^2^). 20% of patients had radiotherapy	60% female for healthy controls and cancer patients	53 ± 16 (healthy control), 56 ± 16 (Cancer patients)	Myocardial T1 mapping was significantly elevated in cancer patients who had received anthracycline treatment compared to the healthy control group
(Melendez et al. 2017)	US	Breast cancer, soft tissue sarcomas, or lymphoma received anthracycline treatment (*n* = 40)	Single-center quasi-experimental study	Anthracycline-based therapy (374.8 ± 0.56 mg/m^2^)	66% women	52 ± 13	A significant elevation of myocardial T1 mapping in patients who received anthracycline
(Muehlberg et al. 2018)	Germany	Sarcoma patients received anthracycline treatment (*n* = 23)	Prospective observational quasi-experimental study	Anthracycline-based therapy (342 ± 23 mg/m^2^)	52% female	58.7 ± 13.4	Non-significant elevation of native T1 mapping from baseline values in either group after completion of therapy
(Tahir et al. 2022)	Germany	Breast cancer women with anthracycline-based therapy (*n* = 39). Among them, *n* = 5 patients received additional trastuzumab. The study also includes breast cancer women with left-sided radiotherapy (*n* = 27)	Prospective cohort quasi-experimental study	Anthracycline-based therapy (360 mg/m^2^, *n* = 34) or radiotherapy (*n* = 27)	100% female	51 ± 11 for patients with anthracycline-based therapy	Only anthracycline-based therapy, resulted in higher myocardial T1 mapping 2 weeks after the completion of treatment. No significant changes were observed 7 months after completing treatment
(van der Velde et al. 2021)	Netherlands	Long-term Hodgkin lymphoma and mediastinal non-Hodgkin lymphoma patients (*n* = 80) and their matched healthy control (*n* = 40)	Single-center quasi-experimental study	Anthracycline-based chemotherapy (*n* = 70, (88% of patients)). All patients were exposed to radiotherapy	46% female for cancer patients. 47% female for healthy controls	47 ± 11 (healthy control), 47 ± 11 (Cancer patients)	A significant elevation of myocardial T1 mapping in long-term lymphoma patients (5 years after being disease free)

References [11–19]

### Quality Assessment of Studies

**Fig. 2:**
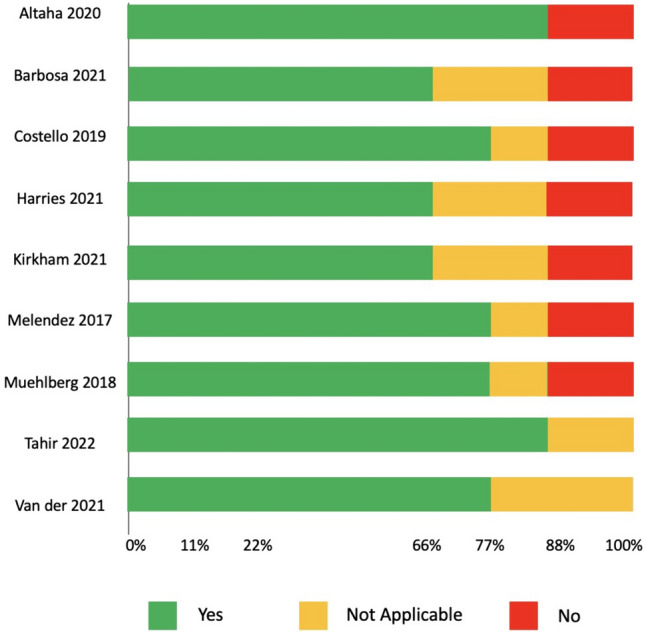
Quality assessment for studies evaluating T1 mapping in patients with anthracycline compared to healthy control or baseline. The Joanna Briggs Institute (JBI) critical appraisal checklist was used to assess the quality of the studies. The JBI quality assessment checklist consists of nine different questions assessing different domains. If a study meets the criteria for a specific question, it gets "Yes," and if it does not meet the criteria, it gets "No." If the study design or methodology is not applicable to the question, it is marked as “Not Applicable.” The percentages of “Yes,” “No,” or “Not Applicable” for each study were calculated based on the proportion of responses relative to the total number of questions. The answers for each question were then color-coded with bars to enhance understanding as follows: “Yes” is represented by green, “No” by red, and “Not Applicable” by yellow

All authors performed the quality assessment independently using the JBI critical appraisal checklist (Fig. 2 and Supplementary Table 4). The JBI quality assessment checklist consists of nine different questions assessing different domains (Supplementary Table 4 and Fig. 2). The nine different questions of the JBI checklist were answered “Yes,” “No,” or “Not applicable,” then the percentages of “Yes”, “No”, or “Not Applicable” for each study were calculated based on the proportion of responses relative to the total number of questions. Notably, most of the items on the JBI checklist were adequately addressed in all studies. The participants included were similar in terms of characteristics such as age, gender, and type of cancer and the intervention involved anthracycline therapy across all studies. In addition, the outcome measure, native myocardial T1 mapping, was consistently assessed across studies through appropriate means using CMR (Fig. 2 and Supplementary Table 4). Overall, these findings reflect the quality and reliability of the included studies for guiding decision-making.

### Elevation of Native Myocardial T1 Mapping in Patients with Anthracycline Compared to Healthy Control

**Fig. 3:**
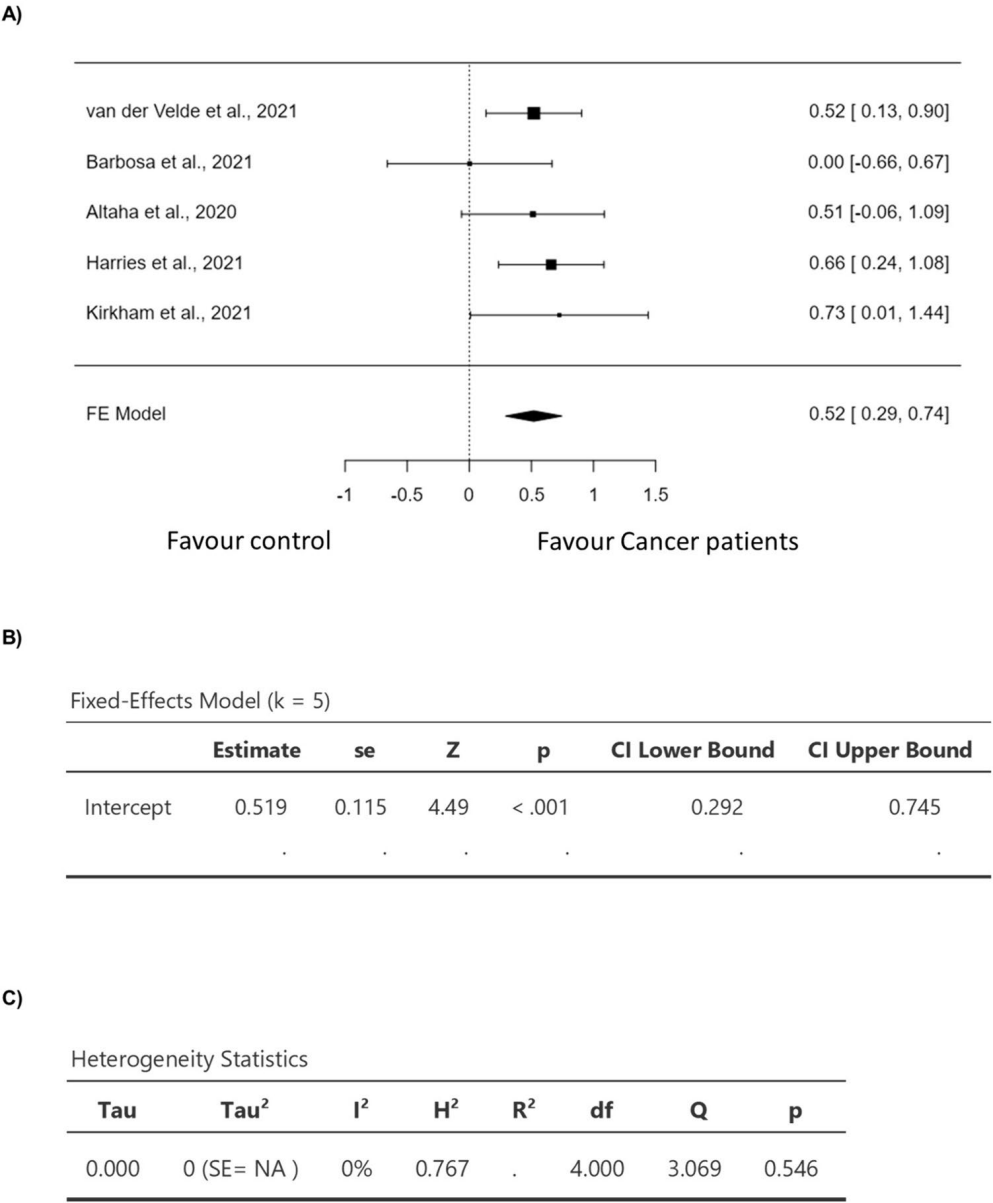
Forest plot for the mean difference in T1 mapping in patients with anthracycline compared to healthy control

**Fig. 4:**
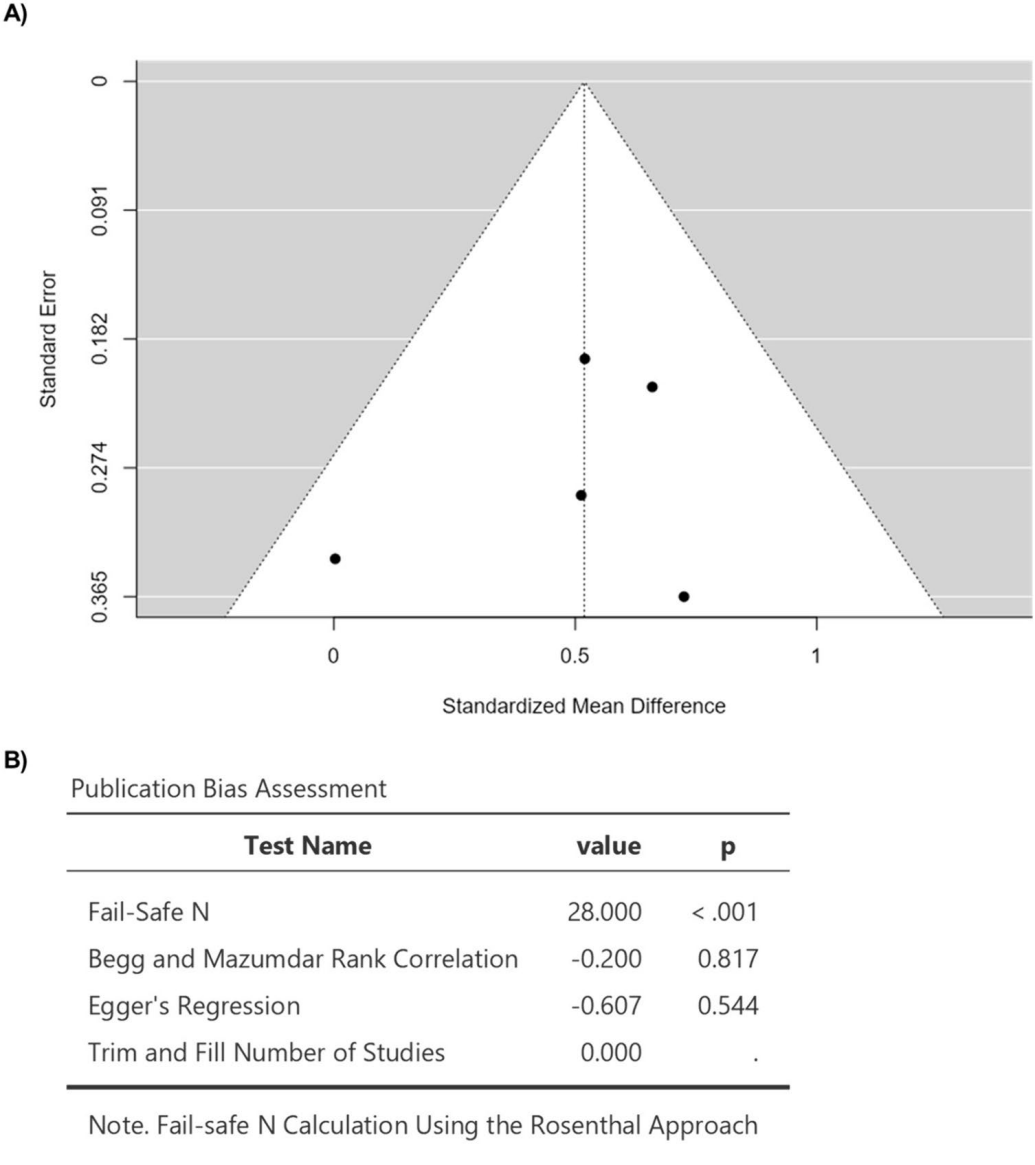
Risk of bias assessment for studies evaluating T1 mapping in patients with anthracycline compared to healthy control

Five studies were included in the analysis (Table 2). The observed standardized mean differences ranged from 0.0029 to 0.7250, with 100% of all estimations being positive (Fig. 3). Based on the fixed-effects model, the estimated average standardized mean difference was 0.5186 (95% CI 0.2925 to 0.7448) (Fig. 3). Hence, the average outcome differed significantly from zero (*z* = 4.4950, *p* < 0.0001) (Fig. 3). The *Q*-test revealed no significant heterogeneity in the true outcomes (*Q*(4) = 3.0691, *p* = 0.5463, *I*^2^ = 0.0000%) (Fig. 3). Analysis of the studentized residuals showed that none of the studies had a value of more than ± 2.5758, indicating the absence of outliers in this model (Fig. 4). Furthermore, based on Cook's distances, no study had an overly influential impact. The rank correlation and regression analyses demonstrated no funnel plot asymmetry (*p* = 0.8167 and *p* = 0.5440, respectively) (Fig. 4). Lastly, sensitivity analysis revealed that removing any study from the analysis had no impact on the statistical significance of the pooled results.

**Table 2:** The effect of anthracycline on myocardial T1 mapping in clinical studies

Study	Healthy controls Mean ± SD	Cancer patients Mean ± SD	T1 mapping method	Time frame
(van der Velde et al. 2021)	964 ± 25 *n* = 40	980 ± 33 *n* = 80	MOLLI	5 years after completing treatment
(Barbosa et al. 2021)	1262.7 ± 31.4 *n* = 17	1262.8 ± 35.5 *n* = 18	MOLLI	88.2 ± 52.1 months after exposure to treatment
(Altaha et al. 2020)	1008.5 ± 23.9 *n* = 30	1022.95 ± 32.8 *n* = 20	MOLLI	3 months after treatment
(Harries et al. 2021)	996 ± 35 *n* = 45	1021 ± 40 *n* = 45	MOLLI	a median interval of 11 (range 3–36) months after completion of anthracycline treatment
(Kirkham et al. 2021)	1212 ± 27 *n* = 16	1231 ± 24 *n* = 16	MOLLI	≥ 3.5 months after completing treatment

References [11, 12, 14, 15, 18]

### Elevation of Myocardial T1 Mapping in Patients Receiving Anthracycline Treatment Compared to Their Baseline Levels Before They Received Anthracycline

**Fig. 5:**
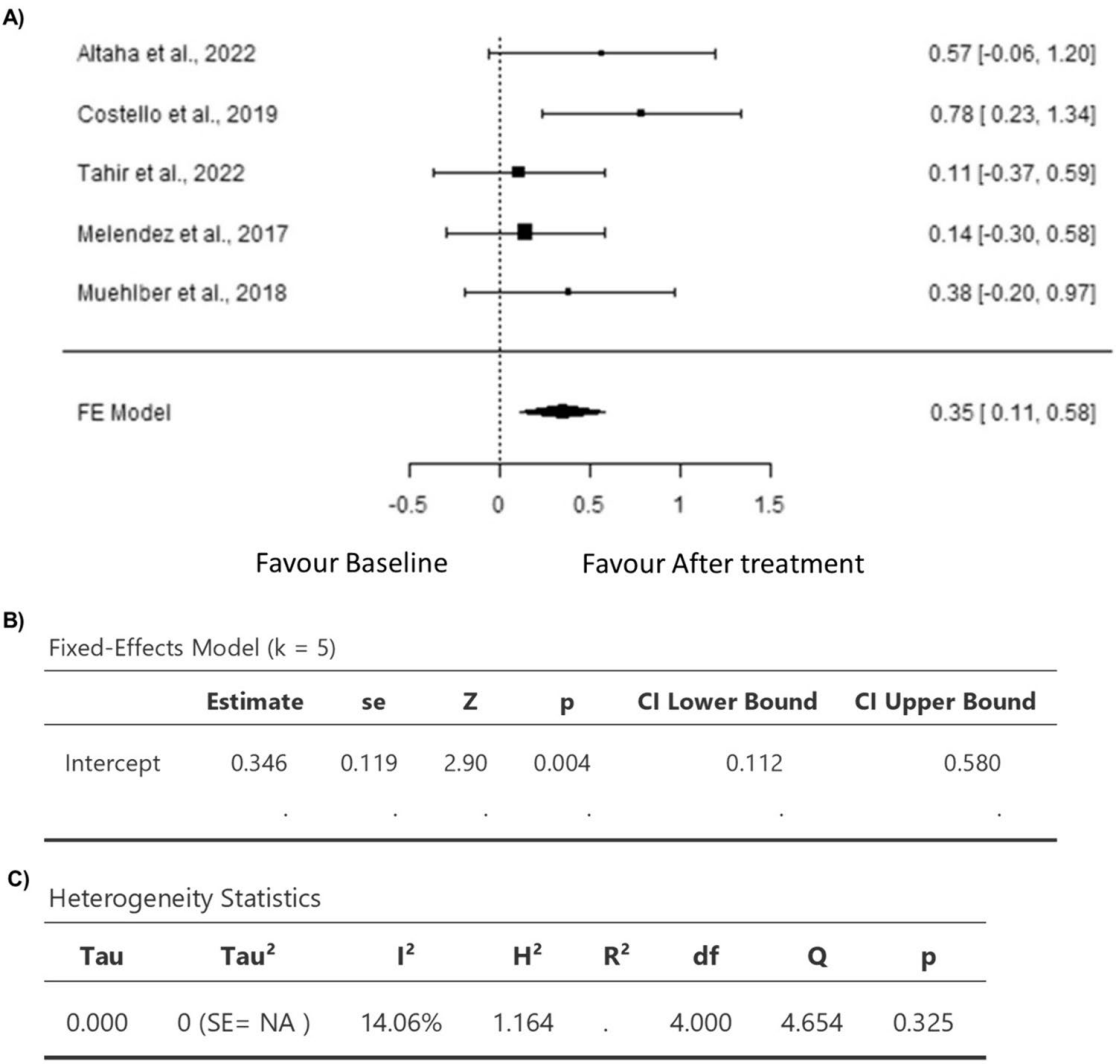
Forest plot for the mean difference in T1 mapping in patients with anthracycline compared to baseline

**Fig. 6:**
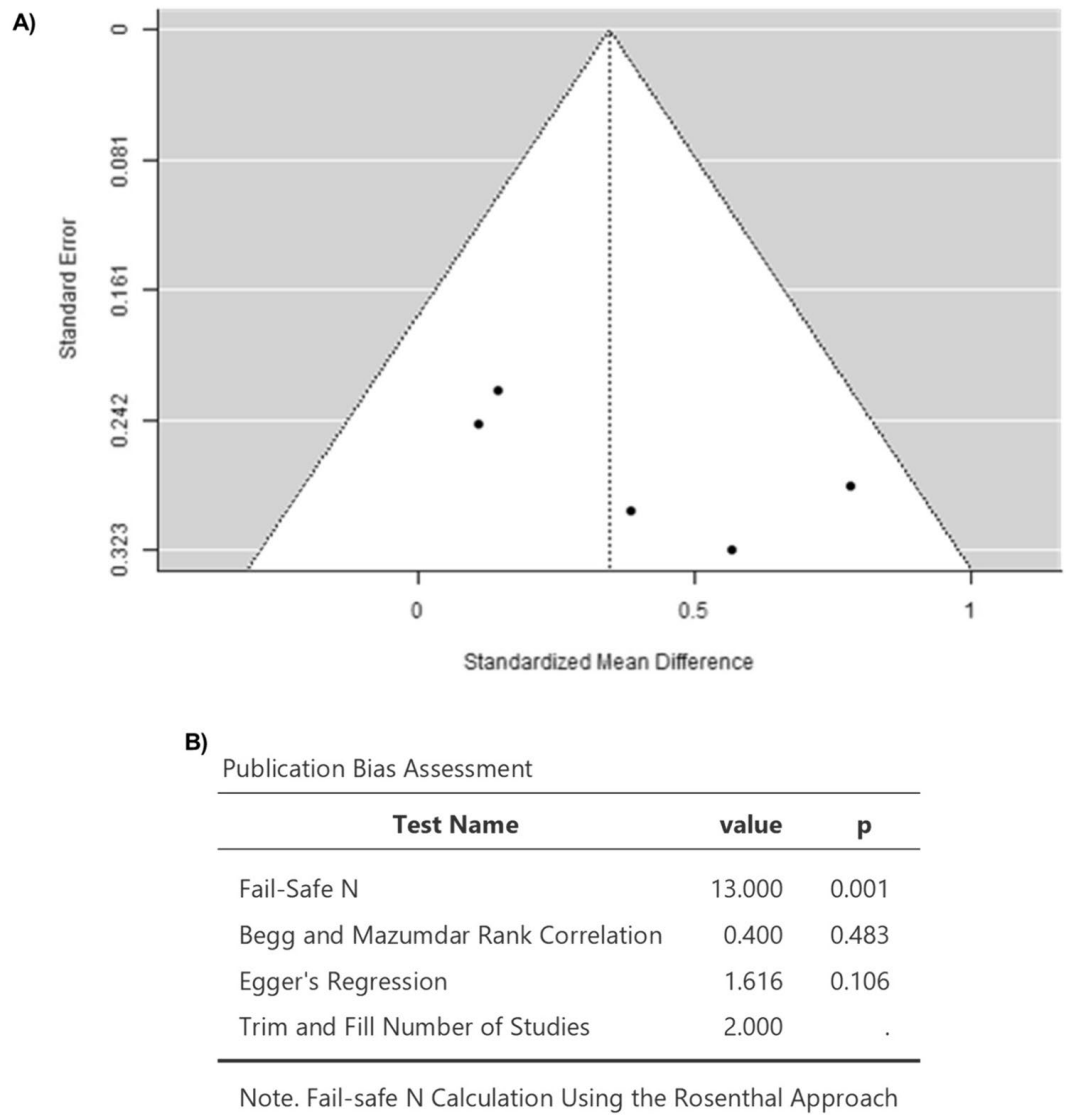
Risk of bias assessment for studies evaluating T1 mapping in patients with anthracycline compared to baseline

A total of *k* = 5 studies were included in the analysis (Table 3). The standardized mean differences varied from 0.1078 to 0.7829, with 100% of all estimations being positive (Fig. 5). Based on the fixed-effects model, the estimated average standardized mean difference was 0.3462 (95% CI 0.1121 to 0.5802) (Fig. 5). Hence, the average outcome differed significantly from zero (*z* = 2.8985, *p* = 0.0037) (Fig. 5). The *Q *test results indicated no significant heterogeneity in the true outcomes (*Q*(4) = 4.6543, *p* = 0.3246, *I*^2^ = 14.0577%) (Fig. 5). The analysis of the studentized residuals showed that none of the studies had a value of more than ± 2.5758, confirming the absence of outliers in this model (Fig. 6). Based on Cook's distances, there was no overly influential study (Fig. 6). Both rank correlation and regression analyses demonstrated no funnel plot asymmetry (*p* = 0.4833 and *p* = 0.1061, respectively) (Fig. 6). Lastly, according to the sensitivity analysis, removing any study from the analysis had no impact on the statistical significance of the pooled results.

**Table 3:** The effect of anthracycline on myocardial T1 mapping in clinical studies

Study	T1 (ms) Before AT Mean ± SD	After AT Mean ± SD	T1 Mapping method	Time frame
(Altaha et al. 2020)	1004.7 ± 28.9 *n* = 20	1022.6 ± 32.8 *n* = 20	MOLLI	At baseline and 3 months after receiving treatment
(Costello et al. 2019)	1174 ± 36 *n* = 27	1203 ± 37 *n* = 27	MOLLI	At baseline and 4 months later (3 weeks after completing treatment
(Tahir et al. 2022)	1245 ± 28 *n* = 34	1248 ± 27 *n* = 33	MOLLI	At baseline and 7 months after completing treatment
(Melendez et al. 2017)	1058.0 ± 100.0 *n* = 40	1071.4 ± 85.2 *n* = 40	MOLLI	At baseline and 3 months after initiating treatment
(Muehlberg et al. 2018)	996 ± 49.9 *n* = 23	1014 ± 41.7 *n* = 23		4 weeks after completing treatment (5 to 6 months after beginning therapy with a mean treatment time of 19.1 ± 2.1 weeks

References [11, 13, 16, 17, 19]

## Discussion

Anthracyclines are very effective anti-cancer agents used in the treatment of several types of cancer, including breast cancer, leukemias, and lymphomas [1]. However, anthracycline therapy triggers cardiotoxicity that may progress to cardiac failure later in life [2]. Thus, using sensitive measures of cardiotoxicity in patients treated with anthracyclines can help in the early detection of cardiac dysfunction and help in initiating interventions to protect these patients [8]. Of those sensitive tools, CMR-measured native myocardial T1 mapping is considered a sensitive and precise quantitative measure of early subclinical cardiac changes, particularly cardiac inflammation and fibrosis [21]. However, the quality and validity of the current evidence supporting the use of these measures in patients treated with anthracyclines is still unclear. Thus, we performed a systematic review and meta-analysis to synthesize results from clinical studies to provide a conclusive answer on the validity of this measure as an early detector of anthracycline-induced myocardial changes. To our knowledge, this study is the first systematic review and meta-analysis to address the quality and the validity of native myocardial T1 mapping in the early detection of anthracycline-induced cardiotoxicity.

In the present study, our systematic review and meta-analysis showed a significant increase in the native myocardial T1 mapping in cancer patients treated with anthracyclines from baseline as well as compared to healthy controls. We also show that this increase in the native myocardial T1 mapping was associated with low heterogeneity among the included studies, suggesting that the outcome is consistent. Given that the LVEF in cancer patients treated with anthracyclines was within the normal range, the native myocardial T1 mapping can likely detect anthracycline-induced myocardial changes before any functional abnormalities. In support of this, recent clinical and preclinical findings demonstrated that native myocardial T1 mapping is a valuable tool for detecting chemotherapy-induced cardiotoxicity early [16, 17]. Thus, our systematic review and meta-analysis suggest that the native myocardial T1 mapping may have a significant clinical utility for the early detection of anthracyclines-induced cardiotoxicity.

In addition to the native T1 mapping, cardiac global longitudinal strain (GLS) is an echocardiographic parameter that can also detect early changes in cardiac function by measuring the longitudinal myocardial deformation [22]. However, GLS values are usually influenced by technical factors, such as image acquisition as well as patient factors, like sex and age [22]. For instance, GLS values are lower in females compared to males and decrease with age in both [22, 23]. Conversely, technical factors like a foreshortening LV image may overestimate the apical strain values [22]. Unlike GLS, the CMR technique, native T1 mapping, quantitatively measures the relaxation time (T1) of myocardial tissue, which reflects the myocardial tissue composition, including the presence of water content (inflammation and edema) and/or collagen (fibrosis) [24]. Thus, it shows diffuse abnormal changes in the cardiac tissue that may precede myocardial dysfunction [24]. Nevertheless, it should be noted that the use of both measures together, GLS and native T1 mapping, may provide a stronger prognostic association with cardiac changes [25].

Given that (1) the native myocardial T1 mapping is a marker of cardiac inflammation, tissue water content, and/or fibrosis [21] and (2) the elevation of the native myocardial T1 mapping occurs before any cardiac functional abnormalities, we speculate that anthracyclines may induce cardiac injury, at least in part, via the activation of cardiac inflammation and fibrosis. This postulation is congruent with the notion that cardiac fibrosis and inflammation play a vital role in cardiotoxicity in several preclinical and clinical studies [26, 27]. In support of this, suppressing inflammation and lessening cardiac fibrosis reduces myocardial injury in numerous pre-clinical models [28–32]. Thus, it is likely that cardiac inflammation and fibrosis may contribute to impaired cardiac and represent a vital mechanism for anthracyclines-induced cardiotoxicity.

While the molecular mechanism of anthracycline-induced cardiac fibrosis and inflammation is still unclear, mounting evidence suggests a pivotal role of nucleotide-binding domain-like receptor protein-3 (NLRP3) inflammasome and myeloid differentiation primary response protein (MyD88) myddosome in the development of cardiac inflammation and fibrosis induced by anthracycline administered either alone or in combination with immune therapy, such as immune checkpoint inhibitors [33, 34]. The activation of NLRP3 inflammasome and MyD88 myddosome increases the secretion of pro-inflammatory cytokines, such as IL-β, thereby contributing to chemotherapy-induced cardiac inflammation and fibrosis [33, 34]. Thus, curtailing the activation of NLRP3 inflammasome and MyD88 myddosome may be a potential therapeutic approach to treat chemotherapy-induced cardiac inflammation and fibrosis. Interestingly, several recent studies have shown that sodium-glucose cotransporter 2 (SGLT2) inhibitors such as empagliflozin or dapagliflozin can reduce cardiac inflammation and fibrosis as well as improve cardiac function by suppressing the activation of NLRP3 inflammasome and MyD88 myddosome [33, 35]. In addition to SGLT2 inhibitors, proprotein convertase subtilisin/kexin type 9 (PCSK9) inhibitors have also demonstrated a potential protective effect against chemotherapy-induced cardiotoxicity by downregulating NLRP3 inflammasome and MyD88 myddosome [36]. Thus, it is likely that strategies that target NLRP3 inflammasome and MyD88 myddosome, such as SGLT2 inhibitors and potentially PCSK9 inhibitors, may hold promise in mitigating anthracycline-induced cardiotoxicity.

An important limitation of this systematic review and meta-analysis is that we have a relatively small number of cancer patients treated with anthracyclines as well as healthy individuals. In addition, the included studies adopted a single-center quasi-experimental design. Thus, multicenter randomized controlled trials with a more significant number of patients and longer-term follow-up are required in order to provide fulsome data about the clinical utility of native T1 mapping for the early detection of anthracycline-induced cardiotoxicity. Also, only non-contrast MOLLI imaging has been used to measure native T1 mapping in the included studies. Nevertheless, non-contrast MOLLI imaging has more clinical feasibility for measuring native T1 mapping over the other imaging tools such as saturation recovery single-shot acquisition (SASHA) due to decreased motion artifact, higher reproducibility, and increased spatial resolution [37]. Lastly, different strength magnets have been used to detect native myocardial T1 mapping suggesting that the meta-analysis is not relatively uniform despite low heterogeneity among the included studies. Given that we do not have access to the raw patient information data, we could not perform a meta-regression analysis.

## Conclusion

In summary, our systematic review and meta-analysis show that the native myocardial T1 mapping is significantly elevated in cancer patients treated with anthracyclines from baseline as well as compared to healthy controls before any myocardial functional abnormalities. We also show that this elevation in the native myocardial T1 mapping was associated with low heterogeneity among the included studies. Thus, our systematic review and meta-analysis suggest that the native myocardial T1 mapping is a valuable tool for detecting anthracycline-induced cardiotoxicity. In addition, given that the native T1 mapping is a marker of cardiac inflammation and fibrosis, and strategies that target cardiac inflammation and fibrosis may help minimize the detrimental effect of anthracyclines on myocardial toxicity.

## Supplementary Information

Below is the link to the electronic supplementary material.

Supplementary file1 (DOCX 35 KB)

Supplementary file2 (DOCX 25 KB)

Supplementary file3 (DOCX 32 KB)

Supplementary file4 (DOCX 21 KB)

## Data Availability

No datasets were generated or analyzed during the current study.
