# Supplementary material for: Assessment of Native Myocardial T1 Mapping for Early Detection of Anthracycline-Induced Cardiotoxicity in Patients with Cancer: a Systematic Review and Meta-analysis
Source: Cardiovasc Toxicol. 2024 May 3;24(6):563–75. doi: 10.1007/s12012-024-09866-1 (PMC11102375; doi:10.1007/s12012-024-09866-1)
Supplement: Supplementary file 1 — Supplementary file1 (DOCX 35 KB) [file 12012_2024_9866_MOESM1_ESM.docx]

**Supplementary Table 1: Search strategy for the electronic databases- the search was done on Thursday, November 10^th^, 2022**

**A.** The search terms used through (PubMed)

| **Concepts** | **Sub-terms** | **Search options** | **Number of hits** |
| --- | --- | --- | --- |
| **1. anthracycline** | 1.1 doxorubicin | MESH term | 63,250 results |
|  | 1.2 idarubicin | MESH term | 1,773 results |
|  | 1.3 anthracycline | MESH term | 76,676 results |
|  | 1.4 chemotherapy | Text Word | 514,183 results |
|  | 1.5 cancer-related treatment | TI and AB | 82 results |
|  | Total 1= 1.1 OR 1.2 OR 1.3 OR 1.4 OR 1.5 | | 551,188 results |
| **2. cancer** | 2.1 cancer* | TI and AB | 2,154,499 results |
|  | 2.2 survivors | TI and AB | 105,878 results |
|  | Total 2= 2.1 OR 2.2 |  | 2,228,437 results |
| **3. CMR** | 3.1 T1 | TI and AB | 123,629 results |
|  | 3.2 Cardiac Magnetic resonance | All field | 63,721 results |
|  | 3.3 CMR | All field | 12,933 results |
|  | Total 3= 3.1 OR 3.2 OR 3.3 |  | 187,029 results |
| **4. cardiotoxicity** | 4.1 cardiotoxicity | MESH term | 3,957 results |
|  | 4.2 myocardial injury | TI and AB | 11,650 results |
|  | 4.3 Heart failure | MESH term | 141,922 results |
|  | 4.4 subclinical systolic function | TI and AB | 5 results |
|  | 4.5 subclinical diastolic function | TI and AB | 547 |
|  | 4.6 Myocardial function | MESH term | 189,836 results |
|  | 4.7 Cardiac involvement | TI and AB | 6,779 results |
|  | Total 4= 4.1 OR 4.2 OR 4.3 OR 4.4 OR 4.5 OR 4.6 OR 4.7 |  | 337,237 results |
|  | Grant Total: |  | 243 results |

**B.** The search used through (Scopus).

| **Concepts** | **Sub-terms** | **Search options** | **Number of hits** |
| --- | --- | --- | --- |
| **1. anthracycline** | 1.1 doxorubicin | TITLE-ABS-KEY |  |
|  | 1.2 idarubicin | TITLE-ABS-KEY |  |
|  | 1.3 anthracycline | TITLE-ABS-KEY |  |
|  | 1.4 chemotherapy | TITLE-ABS-KEY |  |
|  | 1.5 cancer-related treatment | TITLE-ABS-KEY |  |
|  | Total 1= 1.1 OR 1.2 OR 1.3 OR 1.4 OR 1.5 | | 5,697 results |
| **2. cancer** | 2.1 cancer* | TITLE-ABS-KEY |  |
|  | 2.2 survivors | TITLE-ABS-KEY |  |
|  | Total 2= 2.1 OR 2.2 |  | 3,812,798 results |
| **3. CMR** | 3.1 T1 | TITLE-ABS-KEY |  |
|  | 3.2 cardiac magnetic resonance | TITLE-ABS-KEY |  |
|  | 3.3 CMR | TITLE-ABS-KEY |  |
|  | Total 3= 3.1 OR 3.2 OR 3.3 |  | 260,535 results |
| **4. cardiotoxicity** | 4.1 cardiotoxicity | TITLE-ABS-KEY |  |
|  | 4.2 myocardial injury | TITLE-ABS-KEY |  |
|  | 4.3 Heart failure | TITLE-ABS-KEY |  |
|  | 4.4 subclinical systolic function | TITLE-ABS-KEY |  |
|  | 4.5 subclinical diastolic function | TITLE-ABS-KEY |  |
|  | 4.6 Myocardial function | TITLE-ABS-KEY |  |
|  | 5.6 Cardiac involvement | TITLE-ABS-KEY |  |
|  | Total 4= 4.1 OR 4.2 OR 4.3 OR 4.4 OR 4.5 OR 4.6 OR 4.7 |  | 686,082 results |
|  | Grant Total: |  | 958 results |

**C.** The search terms used through (Embase).

| **Concepts** | **Sub-terms** | **Search options** | **Number of hits** |
| --- | --- | --- | --- |
| **1. anthracycline** | 1.1 'doxorubicin':ti,ab | Chemical name | 69,930 results |
|  | 1.2 'idarubicin':ti,ab | Chemical name | 3,372  results |
|  | 1.3 anthracycline:ab,ti | Ti and ab | 20,268 results |
|  | 1.4 chemotherapy:ti,ab | Ti and ab | 678,601 results |
|  | 1.5 cancer-related treatment:ti,ab | Ti and ab | 26,547 results |
|  | Total 1= 1.1 OR 1.2 OR 1.3 OR 1.4 OR 1.5 | | [747,852](https://www.embase.com/) results |
| **2. cancer** | 2.1 cancer*:ti,ab | Ti and ab | 3,016,519 results |
|  | 2.2 survivors:ti,ab | Ti and ab | 154,160 results |
|  | Total 2= 2.1 OR 2.2 |  | [3,120,738](https://www.embase.com/) results |
| **3. CMR** | 3.1 t1:ti,ab | Ti and ab | 149,139 results |
|  | 3.2 cardiac magnetic resonance | Ti and ab | 46,768 results |
|  | 3.3 CMR |  | 25,812 results |
|  | Total 3= 3.1 OR 3.2 OR 3.3 |  | [202,378](https://www.embase.com/) results |
| **4. cardiotoxicity** | 4.1 cardiotoxicity:ti,ab | Ti and ab | 19,986 results |
|  | 4.2 myocardial AND injury:ti,ab | Ti and ab | 52,604 results |
|  | 4.3 heart AND failure:ti,ab | Ti and ab | 440,748 results |
|  | 4.4 subclinical AND systolic AND function:ti,ab | Ti and ab | 3,429 results |
|  | 4.5 subclinical AND diastolic AND function:ti,ab | Ti and ab | 2,817 results |
|  | 4.6 myocardial AND function:ti,ab | Ti and ab | 111,873 results |
|  | 4.7 cardiac AND involvement:ti,ab | Ti and ab | 36,785  results |
|  | Total 4= 4.1 OR 4.2 OR 4.3 OR 4.4 OR 4.5 OR 4.6 OR 4.7 |  | [598,461](https://www.embase.com/) results |
|  | Grant Total: |  | 427 results |

**D.** The search terms used through (Web of Science).

| **Concepts** | **Sub-terms** | **Search options** | **Number of hits** |
| --- | --- | --- | --- |
| **1. anthracycline** | 1.1 AB=(doxorubicin) | AB |  |
|  | 1.2 AB=(idarubicin) | AB |  |
|  | 1.3 AB=(anthracycline) | AB |  |
|  | 1.4 AB=(chemotherapy) | AB |  |
|  | 1.5 AB=(cancer-related treatment) | AB |  |
|  | Total 1= 1.1 OR 1.2 OR 1.3 OR 1.4 OR 1.5 | | 357,059 results |
| **2. cancer** | 2.1 AB=(cancer*) | AB |  |
|  | 2.2 AB=(survivors) | AB |  |
|  | Total 2= 2.1 OR 2.2 |  | 1,748,647 results |
| **3. CMR** | 3.1 AB=(T1) | AB |  |
|  | 3.2 AB=(cardiac magnetic resonance) | AB |  |
|  | 3.3 AB=(CMR) |  |  |
|  | Total 3= 3.1 OR 3.2 OR 3.3 |  | 121,706 results |
| **4. cardiotoxicity** | 4.1 AB=(cardiotoxicity) | AB |  |
|  | 4.2 B=(myocardial injury) | AB |  |
|  | 4.3 AB=(Heart failure) | AB |  |
|  | 4.4 AB=(subclinical systolic function ) | AB |  |
|  | 4.5 AB=(subclinical diastolic function) | AB |  |
|  | 4.6 AB=(Myocardial function ) | AB |  |
|  | 5.6  AB=(Cardiac involvement ) | AB |  |
|  | Total 4= 4.1 OR 4.2 OR 4.3 OR 4.4 OR 4.5 OR 4.6 OR 4.7 |  | 241,617 results |
|  | Grant Total: |  | 152 results |

The total number of studies is 1780 articles.
