# Supplementary material for: Assessment of Native Myocardial T1 Mapping for Early Detection of Anthracycline-Induced Cardiotoxicity in Patients with Cancer: a Systematic Review and Meta-analysis
Source: Cardiovasc Toxicol. 2024 May 3;24(6):563–75. doi: 10.1007/s12012-024-09866-1 (PMC11102375; doi:10.1007/s12012-024-09866-1)
Supplement: Supplementary file 2 — Supplementary file2 (DOCX 25 KB) [file 12012_2024_9866_MOESM2_ESM.docx]

**Supplementary Table 1. The effect of anthracycline on LVEF in clinical studies**

| **Study** | **Healthy Control**  **Mean**  **+ SD** | **Cancer Case**  **Mean**  **+ SD** | **Time Frame** |
| --- | --- | --- | --- |
| (van der Velde et al. 2021) | 60± 5  n = 40 | 53±5  n = 80 | 5 years after completing treatment |
| (Barbosa et al. 2021) | 68.0 ± 4.6  n = 17 | 62.4 ± 7.5  n = 18 | 88.2 ± 52.1 months after exposure to treatment |
| (Altaha et al. 2020) | 60.5 ± 4.2  n =30 | 61.4 ± 4.15  n = 20 | 3 months after treatment |
| (Harries et al. 2021) | 60.8 ± 2.4  n =45 | 59.5 ± 4.1  n =45 | A median interval of 11 (range 3–36) months after completion of anthracycline treatment |
| (Kirkham et al. 2021) | 64 ± 5  n = 16 | 62±4  n = 16 | ≥ 3.5 months after completing treatment |
|  |  |  |  |

Altaha MA, Nolan M, Marwick TH, et al. (2020) Can Quantitative CMR Tissue Characterization Adequately Identify Cardiotoxicity During Chemotherapy?: Impact of Temporal and Observer Variability. JACC Cardiovasc Imaging 13(4):951-962 doi:10.1016/j.jcmg.2019.10.016

Barbosa MF, Fusco DR, Gaiolla RD, et al. (2021) Characterization of subclinical diastolic dysfunction by cardiac magnetic resonance feature-tracking in adult survivors of non-Hodgkin lymphoma treated with anthracyclines. BMC Cardiovasc Disord 21(1):170 doi:10.1186/s12872-021-01996-6

Harries I, Berlot B, Ffrench-Constant N, et al. (2021) Cardiovascular magnetic resonance characterisation of anthracycline cardiotoxicity in adults with normal left ventricular ejection fraction. Int J Cardiol 343:180-186 doi:10.1016/j.ijcard.2021.08.037

Kirkham AA, Paterson DI, Haykowsky MJ, et al. (2021) Aerobic Fitness Is Related to Myocardial Fibrosis Post-Anthracycline Therapy. Med Sci Sports Exerc 53(2):267-274 doi:10.1249/MSS.0000000000002469

van der Velde N, Janus CPM, Bowen DJ, et al. (2021) Detection of Subclinical Cardiovascular Disease by Cardiovascular Magnetic Resonance in Lymphoma Survivors. JACC CardioOncol 3(5):695-706 doi:10.1016/j.jaccao.2021.09.015
