# Supplementary material for: Assessment of Native Myocardial T1 Mapping for Early Detection of Anthracycline-Induced Cardiotoxicity in Patients with Cancer: a Systematic Review and Meta-analysis
Source: Cardiovasc Toxicol. 2024 May 3;24(6):563–75. doi: 10.1007/s12012-024-09866-1 (PMC11102375; doi:10.1007/s12012-024-09866-1)
Supplement: Supplementary file 3 — Supplementary file3 (DOCX 32 KB) [file 12012_2024_9866_MOESM3_ESM.docx]

**Supplementary Table 2. The effect of anthracycline on LVEF in clinical studies**

| **Study** | **Before**  **Mean**  **+ SD** | **After**  **Mean**  **+ SD** | **Time Frame** |
| --- | --- | --- | --- |
| (Altaha et al. 2020) | 63.5 ± 3.6  n = 20 | 61.1 ± 4.15  n = 20 | At baseline and 3 months after receiving treatment |
| (Costello et al. 2019) | 63.1±4.3  n = 27 | 61.3±3.5  n = 27 | 3 weeks after completing treatment |
| (Tahir et al. 2022) | 60 ±4  n = 34 | 59 ±7  n =33 | 7 months after completing treatment |
| (Melendez et al. 2017) | 62 ± 7  n = 40 | 58 ± 7  n = 40 | At baseline and 3 months after initiating treatment |
| (Muehlberg et al. 2018) | 61.35 ± 8.9  n = 23 | 54.1 ± 6.8  n = 23 | 4 weeks after completing treatment (5 to 6 months after beginning of therapy) |
|  |  |  |  |

Altaha MA, Nolan M, Marwick TH, et al. (2020) Can Quantitative CMR Tissue Characterization Adequately Identify Cardiotoxicity During Chemotherapy?: Impact of Temporal and Observer Variability. JACC Cardiovasc Imaging 13(4):951-962 doi:10.1016/j.jcmg.2019.10.016

Costello BT, Roberts TJ, Howden EJ, et al. (2019) Exercise Attenuates Cardiotoxicity of Anthracycline Chemotherapy Measured by Global Longitudinal Strain. JACC CardioOncol 1(2):298-301 doi:10.1016/j.jaccao.2019.09.002

Melendez GC, Jordan JH, D'Agostino RB, Jr., Vasu S, Hamilton CA, Hundley WG (2017) Progressive 3-Month Increase in LV Myocardial ECV After Anthracycline-Based Chemotherapy. JACC Cardiovasc Imaging 10(6):708-709 doi:10.1016/j.jcmg.2016.06.006

Muehlberg F, Funk S, Zange L, et al. (2018) Native myocardial T1 time can predict development of subsequent anthracycline-induced cardiomyopathy. ESC Heart Fail 5(4):620-629 doi:10.1002/ehf2.12277

Tahir E, Azar M, Shihada S, et al. (2022) Myocardial injury detected by T1 and T2 mapping on CMR predicts subsequent cancer therapy-related cardiac dysfunction in patients with breast cancer treated by epirubicin-based chemotherapy or left-sided RT. Eur Radiol 32(3):1853-1865 doi:10.1007/s00330-021-08260-7
