# Supplementary material for: Assessment of Native Myocardial T1 Mapping for Early Detection of Anthracycline-Induced Cardiotoxicity in Patients with Cancer: a Systematic Review and Meta-analysis
Source: Cardiovasc Toxicol. 2024 May 3;24(6):563–75. doi: 10.1007/s12012-024-09866-1 (PMC11102375; doi:10.1007/s12012-024-09866-1)
Supplement: Supplementary file 4 — Supplementary file4 (DOCX 21 KB) [file 12012_2024_9866_MOESM4_ESM.docx]

|  |  | **Altaha 2020** | **Barbosa 2021** | **Costello 2019** | **Harries 2021** | **Kirkham 2021** | **Melendez 2017** | **Muehlberg 2018** | **Tahir 2022** | **Van der 2021** |
| --- | --- | --- | --- | --- | --- | --- | --- | --- | --- | --- |
| **Q1** | **Is it clear in the study what is the ‘cause’ and what is the ‘effect’ (i.e. there is no confusion about which variable comes first)?** | Y | Y | Y | Y | Y | Y | Y | Y | Y |
| **Q2** | **Were the participants included in any comparisons similar?** | Y | Y | Y | Y | Y | Y | Y | Y | Y |
| **Q3** | **Were the participants included in any comparisons receiving similar treatment/care, other than the exposure or intervention of interest?** | N | N | N | N | N | N | N | Y | Y |
| **Q4** | **Was there a control group?** | Y | Y | NA | Y | Y | Y | NA | NA | Y |
| **Q5** | **Were there multiple measurements of the outcome both pre and post the intervention/exposure?** | Y | NA | Y | NA | NA | NA | Y | Y | NA |
| **Q6** | **Was follow up complete and if not, were differences between groups in terms of their follow up adequately described and analyzed?** | Y | NA | Y | NA | NA | Y | Y | Y | NA |
| **Q7** | **Were the outcomes of participants included in any comparisons measured in the same way?** | Y | Y | Y | Y | Y | Y | Y | Y | Y |
| **Q8** | **Were outcomes measured in a reliable way?** | Y | Y | Y | Y | Y | Y | Y | Y | Y |
| **Q9** | **Was appropriate statistical analysis used?** | Y | Y | Y | Y | Y | Y | Y | Y | Y |
|  | **% Yes** | **88%** | **66%** | **77%** | **66%** | **66%** | **77%** | **77%** | **88%** | **77%** |
|  | **% No** | **11%** | **11%** | **11%** | **11%** | **11%** | **11%** | **11%** | **0%** | **0%** |
|  | **%NA** | **0%** | **22%** | **11%** | **22%** | **22%** | **11%** | **11%** | **11%** | **22%** |

*Y: Yes, N: No, NA: Not applicable
